# Treatment of angiomatoid fibrous histiocytoma after unplanned excision: a case report

**DOI:** 10.1186/s13104-018-3736-4

**Published:** 2018-08-31

**Authors:** Kazuhiko Hashimoto, Shunji Nishimura, Ryosuke Kakinoki, Masao Akagi

**Affiliations:** Department of Orthopedic Surgery, Kindai University Hospital, 377-2 Ohno-Higashi, Osaka-Sayama, Osaka 589-8511 Japan

**Keywords:** Unplanned resection, Angiomatoid fibrous histiocytoma, Subcutaneous, Iliac region

## Abstract

**Background:**

Angiomatoid fibrous histiocytoma (AFH) is a relatively uncommon soft tissue tumor of intermediate biologic potential. It occurs in subcutaneous regions of the extremities or the trunk, usually presenting in children or young adults. This is the first reported case of subcutaneous AFH that developed in the iliac region and was treated with an unplanned resection.

**Case presentation:**

An 11-year-old girl noticed a small subcutaneous nodule in the iliac region. As the nodule was asymptomatic, it was observed naturally for a year, after which her parents consulted her doctor due to gradual growth of the nodule. The tumor was resected marginally without biopsy by a non-specialized surgeon. Based on the histology of the resected specimen, the tumor was suspected to be a sarcoma. The patient was referred to our hospital where we reinvestigated the histology of the tumor using immunohistochemistry. After confirming diagnosis of the tumor as an AFH, we undertook additional extensive resection in the iliac region where the tumor had developed. There was no evidence of tumor residue in the resected specimen. It has been 3 years since the operation, and there has been no evidence of recurrence.

**Conclusion:**

We treated a case of AFH after unplanned resection. If subcutaneous tumors in the iliac region are detected, a diagnosis of AFH should be considered and a simple resection avoided.

## Background

Angiomatoid fibrous histiocytoma (AFH) was initially described as angiomatoid malignant fibrous histiocytoma by Enzinger [1]. AFH often presents as a mass in the subcutaneous region in the extremities of children and young adults, with a mean age of approximately 30 years (range 2 months–71 years) [1, 2]. The precise line of differentiation for AFH remains unknown, but it is no longer regarded as malignant because of its benign appearance and favorable prognosis. In the 2013 World Health Organization (WHO) classification, AFH was placed in the category of “intermediate tumors of uncertain differentiation” [3]. Clinically or histologically, it is difficult to differentiate this neoplasm from vascular tumors, such as angiosarcoma or hemangioendothelioma. Although the prognosis of patients with AFH is generally good, it recurs in up to 15% of cases and metastasizes in less than 1% of cases [4, 5]. Therefore, AFH is often resected with wide margins [6]. In some instances, AFH or other tumors that often arise in subcutaneous regions may be treated by unplanned resection [7]. This is the first case report to describe treatment of subcutaneous AFH in the iliac region with additional extended resection after an initial unplanned resection.

## Case presentation

**Fig. 1:**
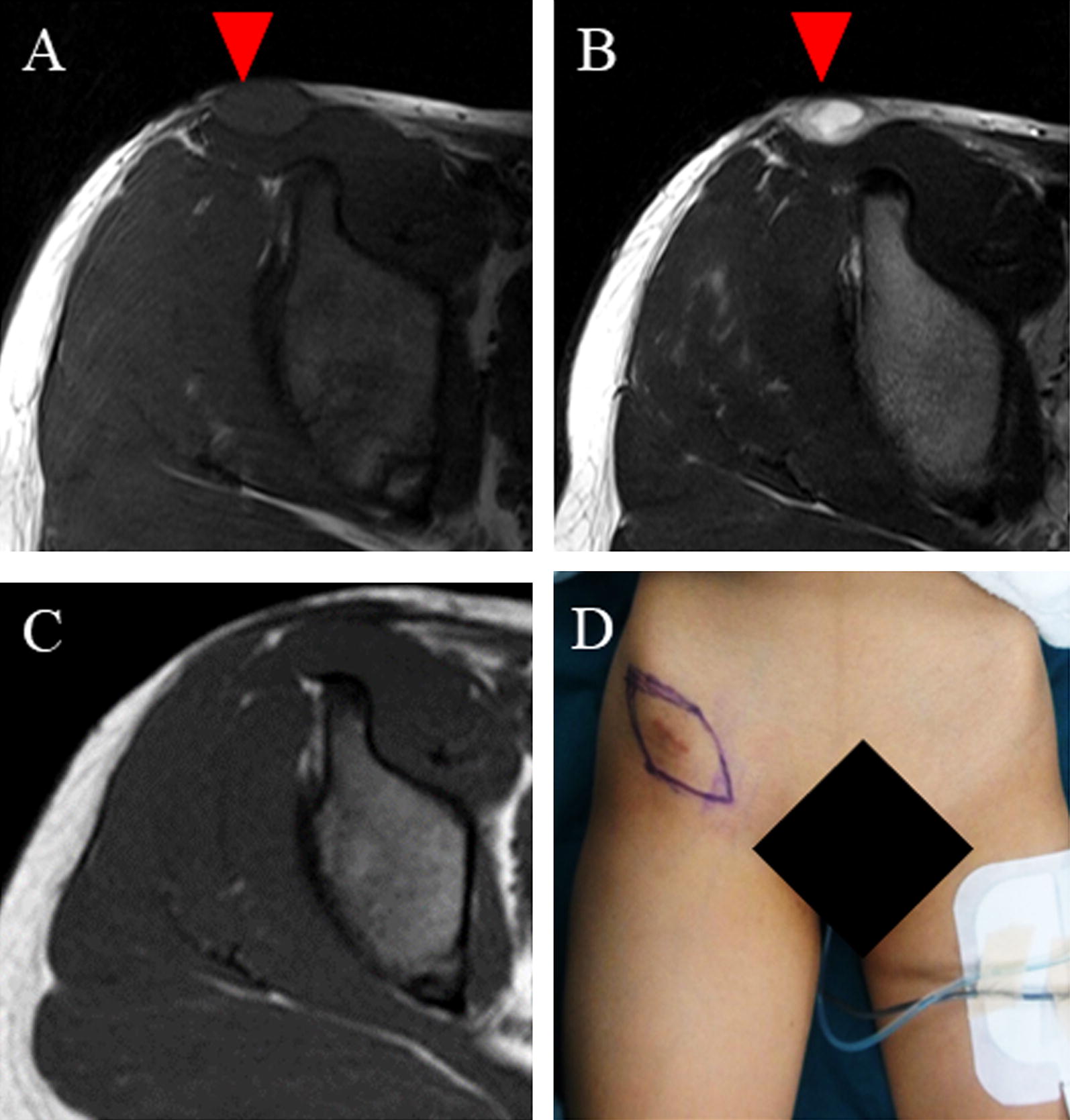
**A** Coronal image of the iliac region acquired using T1-weighted magnetic resonance imaging (MRI). **B** Coronal image of the iliac region acquired using T2-weighted MRI. **C** Coronal image of the iliac region acquired using T2-weighted MRI after unplanned resection. Red arrowheads indicate the tumor. **D** Visual appearance of the iliac region

**Fig. 2:**
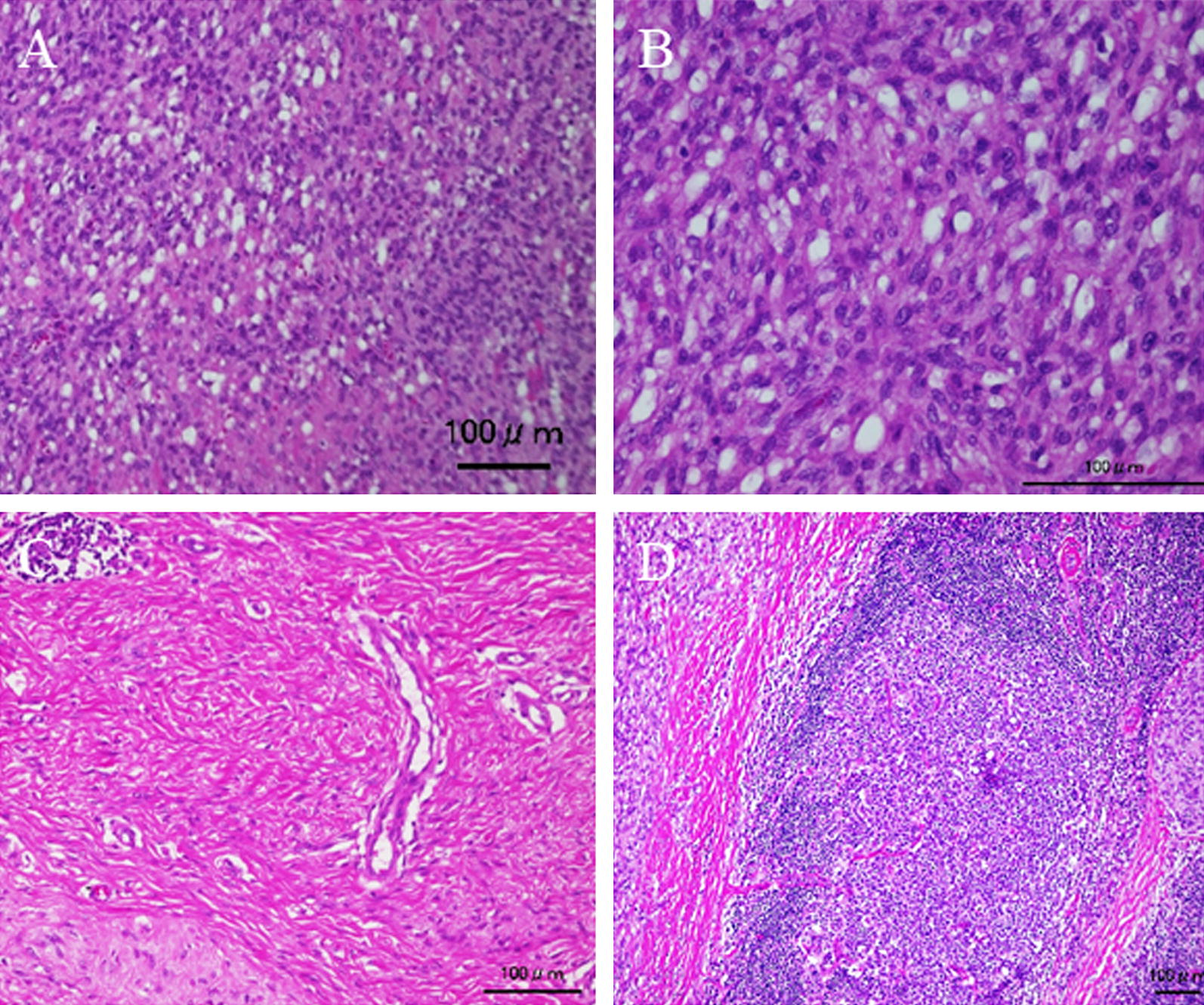
**A** Hematoxylin–eosin (H–E) staining features under ×100 magnification. **B** H–E staining features under ×400 magnification. **C** Pseudo-vessel tissues in the tumor with H–E staining (×200 magnification). **D** Lymph nodes around the tumor with H–E staining (×100 magnification). Scale bar = 100 μm

**Fig. 3:**
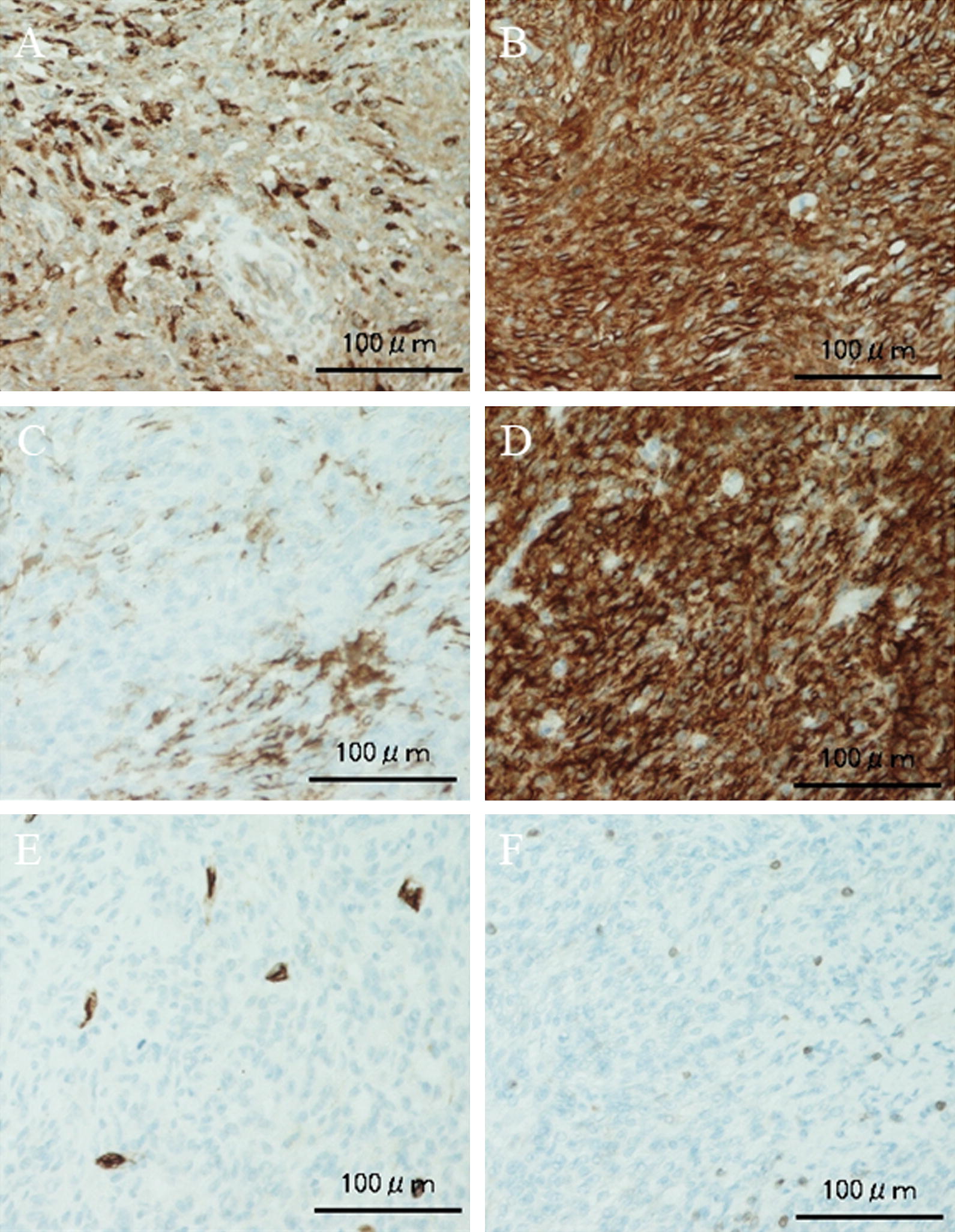
Immunohistochemical staining for **A** CD68, **B** CD99, **C** CD56, **D** epithelial membrane antigen (EMA), **E** CD34 and **F** Bcl-2. CD68 expression is positive (**A**), whilst expression of both CD99 (**B**) and EMA (**D**) is strongly positive. Expression of CD56 (**C**) is focally positive. Expression of CD34 (**E**) is positive only at the site of the blood vessels. Expression of Bcl-2 (**F**) is positive only at the lymph nodes. Scale bar = 100 μm

**Fig. 4:**
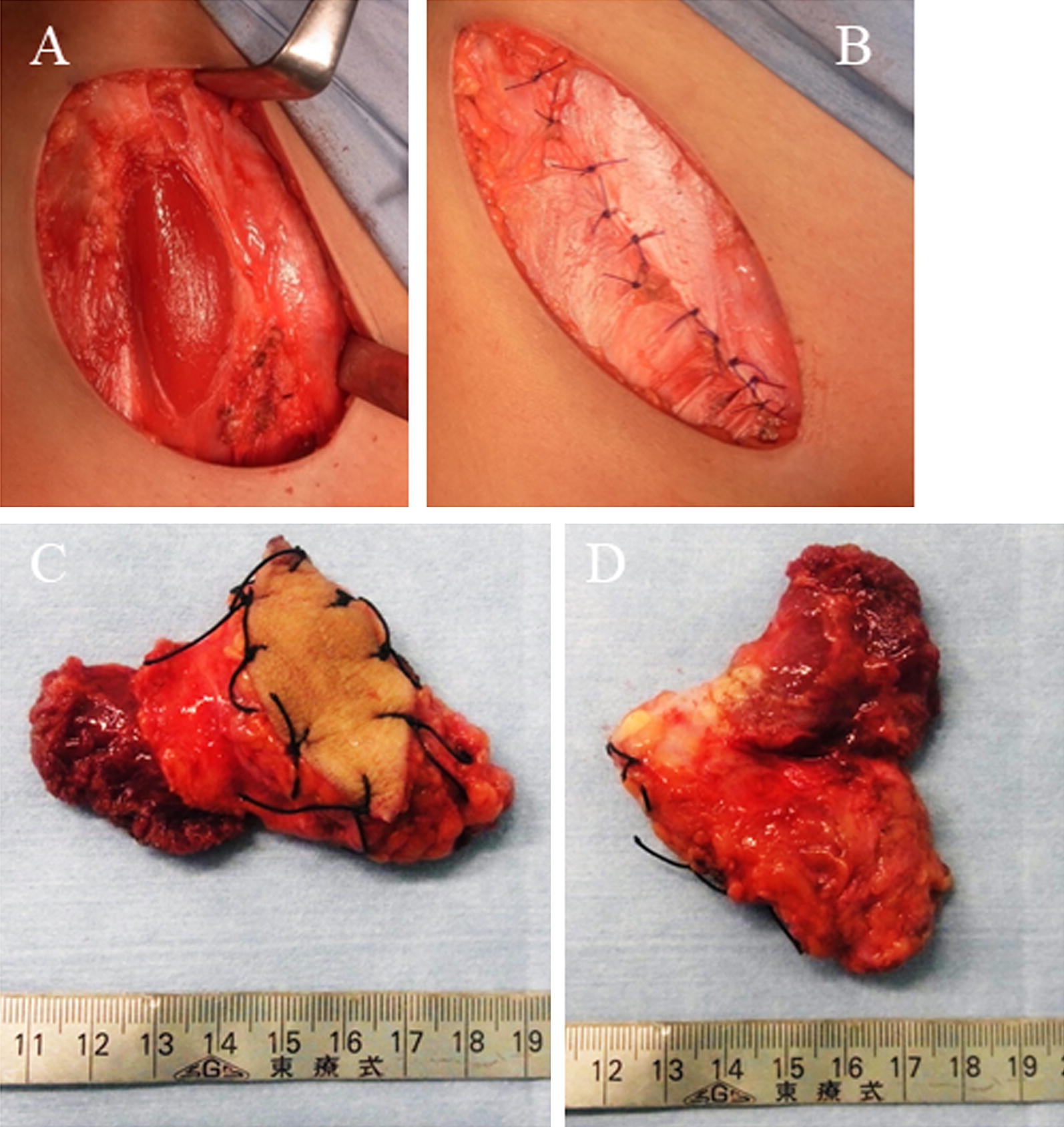
**A** Surgical site after resection of the tumor. **B** The visual appearance of the sutured fascia of the iliac muscle. **C** The visual appearance of the front side of the resected specimen. **D** The visual appearance of the posterior side of the resected specimen. There is no macroscopic residue of the tumor (**C**, **D**)

An 11-year-old girl noticed a small subcutaneous nodule in her iliac region. As the nodule was asymptomatic, she did not consult a doctor for 1 year. After the nodule began to gradually increase in size, she visited a nearby hospital where T1-weighted magnetic resonance imaging (MRI) revealed a 4.3- × 4.1-cm tumor in the subcutaneous iliac region (Fig. 1A). A T2-weighted image showed the tumor with high intensity in the inner cystic region (Fig. 1B). A non-specialized surgeon undertook an unplanned marginal resection, after which MRI showed no evidence of tumor residue (Fig. 1C). An approximately 4-cm scar from surgery was observed in her iliac region (Fig. 1D). As the resected specimen displayed sarcoma-like features on histological examination, she was referred to our hospital. She displayed no symptoms, and her blood test results were normal (Hb = 12.7 g/dl, CRP = 0.019 mg/dl). In our hospital, the histological specimen was investigated again using hematoxylin–eosin (H–E) and immunohistochemical staining. H–E staining showed proliferation of spindle-shaped cells with enlarged nuclei, with stroma composed of fibrous tissue (Fig. 2A, B). Furthermore, we noted the presence of pseudoangiomatous spaces filled with blood and surrounded by tumor cells (Fig. 2C), in addition to lymph nodes surrounding the tumor (Fig. 2D). Immunohistochemical findings revealed positive staining for CD68, CD99, CD56 (focally), and epithelial membrane antigen (EMA) (Fig. 3A–D). Staining for CD34 was positive only in the blood vessels, whilst staining for Bcl-2 was only focally positive within the lymph nodes (Fig. 3E, F). Staining for S-100 was negative (data not shown). On the basis of the aforementioned histological features, we diagnosed AFH. Based on this diagnosis, we undertook an additional extended resection. Skin and one layer of the underlying iliac muscle was resected (Fig. 4A). Because the tumor involved the lateral femoral cutaneous nerve, we had no choice but to excise the nerve clumping with the tumor. We performed closure of the membrane of the iliac muscle (Fig. 4B). There was no evidence of tumor on macroscopic examination of the resected specimen (Fig. 4C, D), which was confirmed by histological examination (data not shown). It has been 3 years since treatment, and the patient has remained disease-free. However, perceptual disorders of the femoral front have remained.

## Discussion and conclusion

Angiomatoid fibrous histiocytoma may arise in any region of the body, including the head, neck, trunk, and lung, but most commonly arises in the extremities [2, 6, 8, 9]. It also occasionally arises in subcutaneous regions, so non-specialized surgeons often undertake unplanned resection [7]. We present the first case of subcutaneous AFH in the iliac region treated with a previous improper resection by a non-specialized surgeon.

The onset age of the current case was similar to that of previously described cases [10]. Subcutaneous AFH in the iliac region is extremely rare. According to previous reports, it is possible that it originates from the fibroblastic reticulum of the interstitium of normal lymph nodes [2, 4]. In the current case, it is possible that the origin of the tumor was the external iliac lymph node. The presence of systemic symptoms, such as weight loss, malaise, fever, and anemia [10–12], which occur in some patients, can aid diagnosis. However, in the current case, the patient experienced no systemic symptoms. Some groups have reported MRI features of AFH, including cystic areas, pseudocapsules, edema of surrounding mass, enhancement, and fluid–fluid levels [10–12], which were observed in the current case. Although the MRI features of AFH described in the literature may aid diagnosis [6, 13], none of them are considered to be significant [10]. The characteristic histological features of AFH have been well described [2]. This includes the following features: (i) multinodular growth of myoid spindle or histiocytoid cells with a distinctive syncytial appearance, (ii) pseudoangiomatous spaces filled with blood and surrounded by tumor cells, (iii) a thick fibrous pseudocapsule with prominent hemosiderin deposition, and (iv) peritumoral lymphoplasmacytic cuffing with occasional germinal center formation. Other immunohistochemical features of AFH include the fact that approximately half of AFH tumors have desmin expression, which may be diffuse or focal, and occasionally exhibit expression of other markers of myoid differentiation such as smooth muscle actin, calponin, and rarely h-caldesmon [2]. Reports on the expression of epithelial membrane antigen, CD99, and CD68 have varied [2–5]. AFH is also indicated by the negative expression of certain markers, which often include skeletal muscle markers, such as myogenin and MyoD, vascular endothelial markers, such as CD31 and CD34, factor VIII-related antigen, CD35, S-100 protein, and cytokeratins [2]. For differential diagnosis, it is often necessary to differentiate AFH from synovial sarcoma because the current histology revealed proliferation of spindle cells. We ruled out a diagnosis of synovial sarcoma based on H–E staining features and negative immunostaining for S-100 and CD34. AFH should also be differentially diagnosed from other soft tissue tumors like aneurysmal benign fibrous histiocytoma, palisaded lymph node myofibroblastoma, and follicular dendritic cell sarcoma [14]. We could differentiate AFH from those tumors by H–E staining features without immunohistochemistry. In the current case, a confirmative diagnosis of AFH was provided by comprehensive analysis. We were unable to examine the presence of fusion genes that have been associated with AFH. EWSR1-CREB1 is the most frequently described gene fusion to date, having been described in more than 90% of cases [15, 16], although EWSR1-ATF1 appears to be more common in AFH that occurs in extra-somatic soft tissue sites [15–17]. FUS-ATF1 has been described least commonly [17].

The prognosis of AFH is generally considered to be favorable [2, 7, 8]. A previous study showed that recurrence occurred in 25% of cases, metastasis occurred in 5%, and death occurred in 5% [18]. However, another study reported that 63% and 21% of patients had local recurrence and metastasis, respectively, and that 12% of patients died due to the disease [19]. In general, soft tissue tumors, particularly sarcomas, should be considered for treatment by a specialized surgeon [20]. Furthermore, extended resection of AFH is recommended because the tumor is considered intermediate [6]. Additionally, if an unplanned resection has been undertaken, the sarcoma should be treated with additional extended resection [21], as was performed in the current study. With such treatment, functional disorder will occur after surgery [22]. In our patient, perceptual disorders of the femoral front remained.

There were some limitations in the current study. First, we did not perform immunohistochemistry for desmin, which is often useful for differential diagnosis. However, we could make a diagnosis without desmin in the current case. Second, we did not examine fusion genes, although we could confirm the diagnosis of AFH without such an analysis. In conclusion, treating clinicians should consider the possibility of AFH when presented with a subcutaneous mass to avoid unplanned resection.

## Abbreviations

AFH: angiomatoid fibrous histiocytoma
EMA: epithelial membrane antigen
MRI: magnetic resonance imaging
Hb: hemoglobin
CRP: c-reactive protein
